# Epidemic spread of ST1-MRSA-IVa in a neonatal intensive care unit, Italy

**DOI:** 10.1186/1471-2431-12-64

**Published:** 2012-06-08

**Authors:** Mario Giuffrè, Domenico Cipolla, Celestino Bonura, Daniela Maria Geraci, Aurora Aleo, Stefania Di Noto, Federica Nociforo, Giovanni Corsello, Caterina Mammina

**Affiliations:** 1 Department of Mother and Child, University of Palermo, I-90127, Palermo, Italy; 2 Department of Sciences for Health Promotion “G. D’ Alessandro”, University of Palermo, Via del Vespro 133, I-90127, Palermo, Italy; 3 PhD School in Food and Human Nutrition, University of Palermo, I-90127, Palermo, Italy

**Keywords:** CA-MRSA, NICU, Epidemiology, Infection control

## Abstract

**Background:**

Community-associated methicillin-resistant *Staphylococcus aureus* (CA-MRSA) has recently emerged as an important pathogen in neonatal intensive care units (NICUs). The purposes of this study were to characterize methicillin-resistant isolates from an outbreak in a NICU, to examine the genetic traits and clonality of CA-MRSA, and to review the characteristics and outcomes of the neonatal cases and investigate the routes of entry and transmission of the MRSA outbreak strain in the NICU under study.

**Methods:**

The study NICU practiced an active surveillance program for multidrug-resistant organisms, including weekly cultures for detection of MRSA from nasal swabs among all the admitted neonates. All first isolates from surveillance cultures and all clinical isolates were submitted for susceptibility testing and genotyping. Data from each infant’s medical records were prospectively included in a database, and the clinical features and outcomes of the colonized/infected infants were assessed.

**Results:**

A total of 14 infants were colonized or infected by a strain of ST1-MRSA-IVa between April and August 2011. The CA-MRSA strain appeared to have been introduced to the NICU by an infected infant transferred from another hospital. The outbreak was successfully contained by multifaceted infection control interventions.

**Conclusions:**

The results of this study confirm that NICU is a healthcare setting with a critical permeability to CA-MRSA. Active surveillance including molecular typing can help to detect and monitor the spread of antimicrobial drug-resistant organisms, and thus trigger timely control interventions.

## Background

Community-associated methicillin-resistant *Staphylococcus aureus* (CA-MRSA) has recently emerged as an important pathogen. CA-MRSA was first identified in infection cases with no previous contact with the healthcare system or other well-recognized risk factors, such as recent antimicrobial drug use [1]. However, it has recently become increasingly identified as a healthcare-associated (HA) pathogen [2]. Neonatal intensive care units (NICUs), nurseries and maternity settings are involved in these CA-MRSA events with alarming frequencies, and as many as 12 out of 18 recent outbreaks in healthcare facilities have occurred in these settings, with seven outbreaks affecting NICUs [3].

CA-MRSA outbreaks have usually been traced to the infiltration of a single strain in a healthcare setting [4]. However, their epidemiological patterns are increasingly difficult to understand, as their occurrence and dissemination routes in the community are largely unknown. The boundaries between CA and HA infections have become blurred, casting doubt on some previously defined criteria for distinguishing between CA- and HA-MRSA [5].

CA-MRSA has generally been considered to be susceptible to most non-β-lactam antibiotics, to carry small staphylococcal chromosomal cassette (SCC)*mec* cassettes (types IV or V), and to frequently produce the Panton-Valentine leucocidin (PVL). These attributes are detected in most strains of USA300, the most successful CA-MRSA strain, which is now endemic in the US, but occur infrequently in other geographical areas, such as Europe [4,6]. Nonetheless, some increasingly resistant CA-MRSA strains have been described [4]. Moreover, the light IV and V SCC*mec* types have proven to be peculiar to some epidemic HA-MRSA clones, 4 such as ST22-MRSA-IV (EMRSA-15) [3,5]. Additionally, PVL-negative CA-MRSA strains have been shown to cause not only community infections, but also healthcare outbreaks [3,5]. Consequently, highly discriminative molecular typing systems are needed to understand the epidemiology of MRSA.

In this study, we report an outbreak of infection/colonization by an ST1-MRSA-IVa strain in a NICU in Palermo, Italy. The aim of the study was to characterize the bacterial isolates, determine the genetic traits and clonality of CA-MRSA, and to review the characteristics and outcomes of the neonatal cases and investigate the routes of entry and transmission of the MRSA strain in the NICU under study.

## Methods

### Setting

The NICU of the university teaching hospital (Azienda Ospedaliero-Universitaria Policlinico “P. Giaccone”), Palermo, Italy, admits 250 infants annually, of all gestational ages (mean 37 weeks, range 26–42). This NICU is associated with the regional reference centre for genetic diseases, and therefore has a high prevalence of neonates with malformations (approximately 20%), as well as of admissions of infants born elsewhere (outborn) (approximately 35%). The NICU includes one intensive care room containing of eight beds and one intermediate care room containing an additional eight beds. The average nurse-to-patient ratios are 1:3 and 1:4 in the two sections, respectively.

### MRSA surveillance

A microbiological surveillance screening program for MRSA has been in place since June 2009 for all infants staying in the NICU. Surveillance specimens from the anterior nares were obtained weekly (each Tuesday) and sent for MRSA detection and typing. Sampling at admission was discontinued after the first 6 months because of a low yield rate. All first isolates from surveillance cultures and all clinical isolates, i.e. those from diagnostic samples submitted to the clinical microbiology laboratory, were genotyped and compared weekly. No routine surveillance cultures were obtained from healthcare workers (HCWs). MRSA-colonized infants were labeled, but not physically segregated, after careful consideration of facility design and ward staffing issues. However, cohort care was implemented by designated HCWs. The NICU had experienced endemic circulation of ST22-MRSA-IVA (EMRSA-15) since 2009. Strict infection control measures had been implemented, including contact precautions, reinforcement of hand hygiene and compliance monitoring by direct observation, but they were unable to definitively interrupt the spread of this clone.

A prospective database was routinely maintained for patients from whom multidrug resistant organisms, including MRSA, were isolated. Data from medical records included demographic characteristics, delivery history, complications of prematurity, use of parenteral nutrition and invasive devices, clinical presentation if any, antimicrobial susceptibility pattern of MRSA isolates, therapeutic interventions, and outcome.

For the purpose of this study, any infant who had one or more screening nasal specimens that yielded MRSA was considered to be colonized, and infected cases were considered to be those with a clinical isolate of MRSA from one or more non-nasal or pharyngeal specimens, or clinical signs/symptoms requiring antimicrobial therapy.

The ST1-MRSA outbreak period was defined as the time between admission of the first ST1-MRSA case and the discharge of the last case (April 3 to August 12, 2011). The study protocol was approved by the Ethics Committee of the Azienda Ospedaliero-Universitaria Policlinico “P. Giaccone”, Palermo, Italy, and informed consent was sought in accordance with the principles of the Declaration of Helsinki. Written informed consent was obtained from the parents of each patient.

### Laboratory methods

Surveillance specimens for MRSA culture were obtained with cotton swabs and processed within 4 h. Swabs were incubated overnight in Brain Heart Infusion broth, and then inoculated onto mannitol salt agar, incubated in air at 37°C and examined at 24 and 48 h. Presumptive *S. aureus* isolates were identified according to standard methods [7]. MRSA isolates were searched for by colony screening onto oxacillin agar (Mueller-Hinton with oxacillin 6 mg/L) and confirmed using cefoxitin disks. Susceptibility testing of clinical isolates was carried out using the Phoenix™ Automated Microbiology System (Becton Dickinson Diagnostic Systems, Sparks, MD, US). Antibacterial drug susceptibility of surveillance isolates was routinely performed using the disk diffusion method by determining the susceptibility of each isolate to erythromycin, clindamycin, sulfamethoxazole-trimethoprim, tetracycline, ciprofloxacin, gentamicin, tobramycin, linezolid, rifampicin, vancomycin and teicoplanin [8]. *S. aureus* ATCC 25923 was used as the quality control strain.

### Molecular typing

Polymerase chain reaction (PCR)-based strategies were used to identify the structural type of the SCC*mec* element and the PVL genes (*luk*S-PV, *luk*F-PV) [4,9,10]. Multilocus sequence typing (MLST) was performed, as previously described [11]. The MLST allelic profiles and sequence types were assigned by submission to the *S. aureus* MLST database (http://www.mlst.net).

Genotypic characterization of the MRSA isolates was performed by multilocus variable number tandem repeat analysis (MLVA) [12]. Patterns were digitized and analyzed using Bio-Numerics software (Applied Maths, Ghent, Belgium).

## Results

### Description of the outbreak

**Figure 1:**
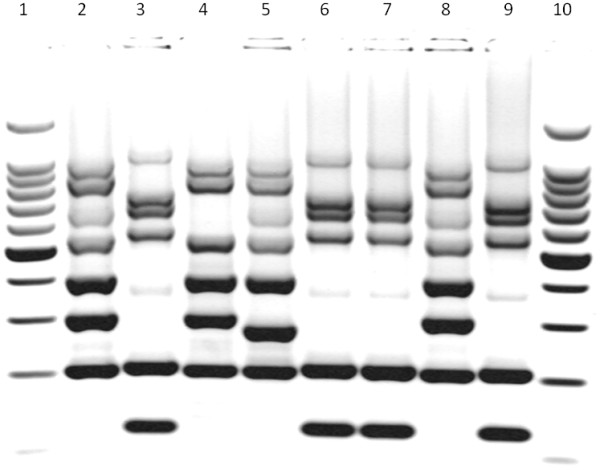
**Multilocus variable number tandem repeat analysis (MLVA) of some representative isolates of methicillin-resistant Staphylococcus aureus (MRSA) identified during the outbreak period in the neonatal intensive care unit (NICU) under study.** Lanes: 1 and 10, 500-bp DNA ladder; 2, 4, 5 and 8, ST22-MRSA-IVa isolates; 3,6,7 and 9, ST1-MRSA-IVa isolates.

Two MRSA isolates were identified from blood and the first surveillance nasal sample of an infant, respectively, on April 5, 2011. These isolates displayed a different drug susceptibility pattern from that of the endemic ST22-MRSA-IVa isolates, and were resistant to gentamicin, erythromycin and clindamycin, unlike the ST22 MRSA isolates. The patient was an extremely-low-birthweight (ELBW) male infant, who had been transferred the previous day from the NICU of another hospital in Palermo, Italy, where he was born on February 19. He required surgery because of an intestinal subocclusion (Table 1). Before admission, the patient was exposed to a high colonization risk because of his prolonged stay in NICU and a previous transfer to a pediatric surgical division. A further indistinguishable isolate was detected in an axillary skin swab cultured on April 26, after the onset of dermatitis. MLVA typing immediately confirmed the introduction of a new strain to the study NICU (Figure 1). He was treated empirically with ampicillin-sulbactam and netilmicin for presumed sepsis, and then with teicoplanin and meropenem after the blood culture yielded MRSA. His clinical condition improved progressively and no further symptoms of MRSA infection had developed up to discharge..

**Table 1:** Characteristics of infants colonized with ST1-MRSA-IVa in an NICU in Palermo, Italy, 2011

**Case**	**Gender**	**Birth date**	**Admission date**	**Inborn/outborn**	**Gestational age at birth (week)**	**Weight at birth (g)**	**Delivery**	**Reason for admission**	**Length of stay (days)**	**Date of first isolation of ST1-MRSA-IVa^1^**	**Risk factor(s)**	**Outcome**
1	F	March 28	April 3	outborn	28	760	CS	surgery	45	May 5	CVC, ET, nCPAP, TPN	discharged
2^2^	M	February 19	April 4	outborn	29	960	CS	surgery	35	April 5	CVC, ET, nCPAP	discharged
3	F	May 9	May 9	outborn	35	2240	CS	respiratory distress	19	May 10	CVC	discharged
4	F	May 16	May 16	inborn	39	2770	CS	malformation	41	June 14	CVC, ET	died
5	F	May 4	May 17	outborn	37	2660	VD	malformation	79^3^	June 28	CVC, ET, nCPAP	discharged
6	M	May 17	May 17	inborn	30	1420	CS	preterm care	42	May 24	CVC, nCPAP	discharged
7	F	June 6	June 6	inborn	39	3090	VD	malformation	49	July 12	CVC	discharged
8	F	June 11	June 11	outborn	27	870	CS	preterm care	62	June 28	CVC, ET, nCPAP, TPN	discharged
9	F	June 12	June 13	outborn	30	1450	CS	preterm care	41	July 5	CVC, ET, nCPAP	discharged
10	M	June 19	June 19	inborn	36	2380	CS	respiratory distress	12	June 28	none	discharged
11	F	June 22	June 22	inborn	33	1360	CS_19_	malformation	26	July 12	CVC, ET, nCPAP, TPN	died
12	F	July 7	July 7	outborn	37	1760	CS	malformation	22	July 12	none	discharged
13	F	July 13	July 13	inborn	39	4220	CS	respiratory distress	7	July 19	none	discharged
14	M	July 17	July 18	inborn	42	1980	VD	respiratory distress	14	July 19	none	discharged

CS = caesarian section, VD = vaginal delivery, CVC = central venous catheter, ET = endotracheal tube, nCPAP = nasal continuous positive airway pressure, TPN = total parenteral nutrition.

^**1**^**weekly screening.**

^**2**^**index case.**

^**3**^**transferred to a cardiac surgery ward after 69 days of NICU stay and re-admitted after 6 days**

On May 5, a second MRSA strain resistant to gentamicin, erythromycin and clindamycin was identified from the nasal swab of another outborn infant who had been admitted the day before the index case (Table 1). This was a female ELBW infant who was admitted to the NICU because of necrotizing enterocolitis with intestinal perforation. She underwent two laparotomy interventions on April 4 and 27, respectively, and was prescribed ampicillin-sulbactam, netilmicin, vancomycin and meropenem. MRSA with characteristics indistinguishable from the nasal swab isolate was detected from the endotracheal tube on May 20, and from the weekly surveillance nasal swab until discharge. Microbiological cultures yielded no further pathogens and her clinical condition gradually improved.

**Figure 2:**
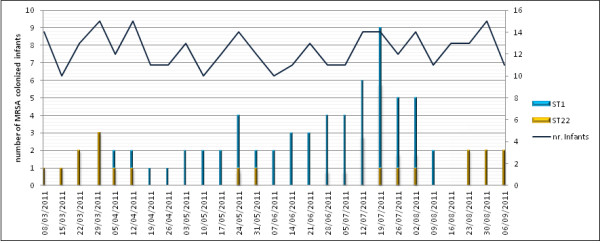
**Epidemic curve of methicillin-resistant Staphylococcus aureus (MRSA)positive cases during the outbreak period.** Curve shows the number of infants whose nasal swabs tested positive for ST1-MRSA-IVa and ST22-MRSA-IVa and the number of hospitalized infants by week.

**Figure 3:**
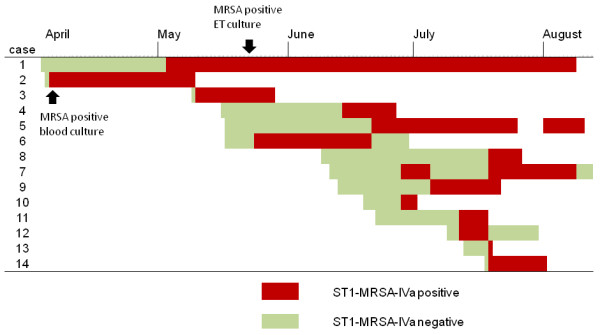
Time course of hospitalization and onset of ST1-MRSA-IVa colonization, as assessed by the weekly surveillance screening program, during the outbreak at the NICU under study.

An increase in the MRSA isolation rate in the NICU was observed between May and August 2011 (Figure 2). A further 12 (15.2%) of 79 neonates who were admitted during the outbreak period were found to be colonized by the new MRSA strain. The temporal distribution of hospitalization and onset of colonization in the 14 cases colonized by ST1-MRSA-IVa, as detected by the weekly active surveillance culture program, is illustrated in Figure 3. No attempt was made to decolonize the infants during the outbreak period. It was also impossible to assess the duration of MRSA colonization, because 10 neonates remained colonized at discharge. Two colonized infants died as a result of malformations.

### Characterization of MRSA

All the MRSA isolates identified by the surveillance screening program during the ST1-MRSA outbreak period were submitted to antibiotic susceptibility testing, PCR for the SCC*mec* element and PVL genes, MLST and MLVA typing. Between one and thirteen isolates were available from each patient, depending on their lengths of stay.

Antimicrobial susceptibility testing of all MRSA isolates from the 14 infants involved in the outbreak indicated resistance to β-lactam antibiotics, gentamicin, erythromycin, clindamycin and tetracycline, but susceptibility to ciprofloxacin, linezolid, rifampicin, sulfamethoxazole-trimethoprim, teicoplanin, tobramycin and vancomycin. All the isolates carried a SCC*mec* cassette type IVa and were negative for the PVL genetic determinants. In addition, MLST demonstrated that they belonged to ST1. MLVA showed that all isolates from the 14 infants had electrophoretic profiles indistinguishable from each other, but quite different from those of the ST22 isolates (Figure 1).

### Infection control measures

Infection control measures were reviewed and reinforced. These included special attention to hand disinfection and environmental cleaning. Anyone caring for an infant infected/colonized by MRSA was required to wear a facemask, gown and gloves. Infected and colonized infants were placed under contact precautions and cohorted in the NICU. All equipment and caring devices in the NICU were thoroughly cleaned and disinfected. All NICU nursing and medical personnel were informed of the outbreak and training sessions on infection control practices and modes of transmission of MRSA were arranged. Outbreak meetings identified the high prevalence of patients admitted to the NICU for surgery or malformations during the outbreak period as the likely major triggering factor, with cross-transmission via the hands of HCWs as the likely major cause of dissemination of the ST1 strain within the NICU. No additional factors were identified. The outbreak was considered ended on August 12, when the last colonized patient was discharged.

## Discussion

MRSA colonization and infection in infants are associated with significant morbidity and economic impact [13]. ELBW infants are particularly fragile individuals who may develop severe diseases. Several possible reservoirs and multiple sources of exposure to MRSA have been reported for infants hospitalized in nurseries and NICUs [14], and transmission of the USA300 strain among postpartum women affected by skin and soft tissue infections has been described [15]. Cases of CA-MRSA transmission associated with breast milk or carriage by a family member have also been reported [16,17]. MRSA skin and soft tissue infections among full-term newborns were detected in the first 30 days since their delivery in US hospitals, as well as in neonates discharged from a nursery [18]. Moreover, previous studies have identified HCWs as reservoirs [19-21].

In the current study, the index case was an infected infant born elsewhere. A second, long-stay, cross-colonized patient likely became the main reservoir of MRSA, while subsequent cross-transmission probably occurred via the hands of staff. MRSA screening at admission and HCW screening were not in place when the index case was admitted, but the weekly surveillance nasal swabs were obtained within 24 h of this patient’s admission, suggesting that ST1-MRSA-IV was imported from another healthcare facility on this occasion. However, alternative or concurrent exposure sources, such as previously colonized infants or HCWs, cannot be entirely ruled out. Indeed, early acquisition of MRSA soon after hospitalization has been described previously [21]. Moreover, because the nares were the only surveillance sampling sites, it is possible that infants colonized at other sites could have escaped detection. In this regard, it has been suggested that sampling sites traditionally associated with HA-MRSA, such as the nose, could lack sensitivity when screening for CA-MRSA colonization [22].

The issue of HCWs as a possible exposure source of MRSA, and especially of CA-MRSA, is widely debated [23]. Although HCWs have been implicated as the source of MRSA in many NICU outbreaks, there are conflicting reports regarding the cost-effectiveness of HCW screening and decolonization [18,19,24]. Evidence of transmission of MRSA from HCWs to patients in an outbreak setting ranges from 5.8–25.5% of cases, with an even lower proportion caused by asymptomatic carriers [25,26]. Moreover, data about the positive impact of HCW screening and decolonization are difficult to assess, because both interventions are usually implemented as a part of a composite pattern of infection control procedures [20,23,27]. Decolonization of staff and patients by mupirocin proved unable to control a NICU outbreak, according to Lepelletier *et al.*[28]. In addition, a decline in the incidence of MRSA infections in hospital over a 7-year surveillance period was shown to persist, despite an interruption to routine HCW screening after the first 4 years [29]. However, the involvement of the HCWs was not assessed in the current study, which represents a substantial limitation. ST1-MRSA carriage by the staff of the NICU was, however, thought to be unlikely the because transmission chain was ultimately interrupted without the adoption of screening and decolonization procedures for HCWs. Nonetheless, emergence of CA-MRSA strains as HA pathogens presents a challenge for MRSA control strategies through the increasingly probable reintroduction of CA-MRSA from the community reservoir [29,30]. Within such an epidemiological framework, HCWs as well as patient’s visitors could play critical roles as potential CA-MRSA traders in NICU outbreaks [3]. Consequently, screening and decolonization of HCWs could be more effective, and should be recommended when dealing with CA-MRSA.

This study had some limitations. As mentioned above, HCWs were not screened either as part of the routine surveillance program or during the outbreak phase. Moreover, infants were not routinely screened at admission, which could have biased the source attribution of the ST1-MRSA-IV strain. Finally, the specific NICU setting and local epidemiology of CA-MRSA make it difficult to generalize from the findings.

## Conclusions

The present investigation showed that traditional approaches for the control of HA-MRSA were also effective in terminating CA-MRSA transmission in the NICU setting. Molecular typing by MLVA helped to clarify the epidemiological features of this CA-MRSA outbreak quickly, by identifying the outbreak ST1-MRSA-IVa strains from the background of the endemic EMRSA-15 colonization cases.

HA transmission of CA-MRSA strains, as well as increasing non-β-lactam resistance in CA-MRSA clones, present increasingly serious challenges to infection control and the clinical management of infections caused by these strains. Monitoring of global and local epidemiologies is critical to assess epidemiological trends and to guide empiric antibiotic options.

## Abbreviations

MRSA, Methicillin-resistant Staphylococcus aureus; CA-MRSA, Community-associated MRSA; ELBW, Extremely-low-birthweight; HA-MRSA, Hospital-associated MRSA; HCW, Healthcare worker; NICU, Neonatal intensive care unit; PCR, Polymerase chain reaction; PVL, Panton-Valentine leucocidin; MLST, Multilocus sequence typing; MLVA, Multilocus variable number tandem repeat analysis; SCCmec, Staphylococcal chromosomal cassette mec.

## Competing interests

The authors declare that they have no competing interests.

## Authors’ contributions

MG, DC, GC and CM designed and supervised the study and drafted the manuscript. SD and FN carried out the study in the field, by participating in the surveillance program, and contributed to the interpretations of results. DMG was in charge of isolation, identification and susceptibility testing. AA and CB were in charge of molecular typing. All authors have read and approved the final manuscript.

## Pre-publication history

The pre-publication history for this paper can be accessed here:

http://www.biomedcentral.com/1471-2431/12/64/prepub
